# The compound role of a coordinator for home-dwelling persons with dementia and their informal caregivers: qualitative study

**DOI:** 10.1186/s12913-020-05913-z

**Published:** 2020-11-16

**Authors:** Stein Erik Fæø, Oscar Tranvåg, Rune Samdal, Bettina S. Husebo, Frøydis K. Bruvik

**Affiliations:** 1 Centre for Elderly and Nursing Home Medicine, Department of Public Health and Primary Care, University of Bergen, P.O. Box 7800, NO-5020 Bergen, Norway; 2 Faculty of Health studies, VID Specialized University, Bergen, Norway; 3 Department of Health and Caring Sciences, Faculty of Health and Social Sciences, Western Norway University of Applied Sciences, Bergen, Norway; 4 Norwegian National Advisory Unit on Women’s Health, Oslo University Hospital, Rikshospitalet, Oslo, Norway; 5 Municipality of Bergen, Norway; 6 Department of Public Health and Primary Care, University of Bergen, Bergen, Norway

**Keywords:** LIVE@Home.Path, Care coordination, Dementia, Home-dwelling, Decision-making, Case management, Empowerment, Qualitative study

## Abstract

**Background:**

As the number of persons with dementia is increasing, there has been a call for establishing sustainable clinical pathways for coordinating care and support for this group. The LIVE@Home.Path trial is a multicomponent, multi-disciplinary intervention combining learning, innovation, volunteer support and empowerment. To implement the intervention, a municipal coordinator has a crucial role. Implementation research on multicomponent interventions is complex and we conducted a qualitative study, aiming to explore the coordinator role and how a coordinator may empower persons with dementia in decision-making processes.

**Methods:**

Qualitative program evaluation combined with a hermeneutic interpretive approach was chosen as methodological approach. Sixteen dyads, consisting of the person with dementia and their main informal caregiver received the intervention by two coordinators. Of these, six dyads, three informal caregivers alone and the two care coordinators along with their leader, in sum, eighteen persons, participated in in-depth or focus group interviews, sharing their experiences after 6 months intervention.

**Results:**

We found that the coordinators fulfilled three functions for the participating dyads: *being a safety net*, meaning that the dyads might have little needs at the moment, but found safety in a relation to someone who might help if the situation should change; *being a pathfinder*, meaning that they supported the dyads in finding their way through the complicated system of care and support services; *being a source for emotional care and support*, meaning that they listened, acknowledged and gave counsel in times of distress. The coordinators emphasized that a trusting leader and work environment was crucial for them to fulfill these functions. We also found that it was challenging for the coordinators to build a relation to the persons with dementia in order to pursue genuine empowerment in decision-making processes.

**Conclusion:**

We found the framework for follow-up to be a feasible starting point for establishing empowering coordination and a sustainable care pathway for persons with dementia and their informal caregivers. More meeting points between coordinator and person with dementia should be pursued in order to fulfill the persons’ fundamental rights to participate in decision-making processes.

**Supplementary Information:**

The online version contains supplementary material available at 10.1186/s12913-020-05913-z.

## Background

As the prevalence of dementia is rapidly increasing, the World Health Organization (WHO) has labeled dementia a global public health priority and urged nations to establish dedicated dementia plans to secure adequate support for this group [1, 2]. In the wake of this call, guidelines and models for developing structured care pathways for persons with dementia has been proposed [3, 4].

Research suggests that persons with dementia living at home may have a high degree of unmet needs ranging from safety issues and general health needs, to legal issues or sustaining meaningful activities. This represents a wide range of needs from a variety of care providers [5]. A review on ‘what works’ to support this group suggests that multicomponent flexible interventions is necessary in order to meet the complex needs of home dwelling persons with dementia. Additionally, intervention studies aiming to support this group often lack the patients’ perspectives and fail to acknowledge the complexity in the patients’ life situation [6]. Persons with dementia are depending on a high degree of informal care which in turn represents a potential health risk for their informal caregivers [7]. To meet the diversity of needs within this group, various approaches of case management and care coordination have emerged. Many of these attempts have proved beneficial, but not uniformly across studies. A Cochrane review [8] found that case management approaches in care for home-dwelling persons with dementia might lead to reduced institutionalization and decreased burden and depression among caregivers. However, the results were inconsistent throughout repeated measurements. Similarly, a meta-analysis of research on ‘coordinating interventions’ in community based dementia care, found significant effects in terms of reduced neuropsychiatric symptoms and caregiver burden [9]. Still, high costs and vaguely defined tasks combined with a multitude of different intervention designs and outcome measures leaves the initiatives fragile and general implementation in practice has been absent. Iliffe [10] suggests ‘fluidity’ as a concept to build further research on these matters upon – one should attempt to establish a base of necessary components for care coordination to function and share experiences of how these components may be adapted to fit in various contexts. As there are various terms for the varieties within this concept, we will refer to the term used by the authors when referring to other publications. In our study we use the terms ‘care coordination’ and ‘coordinator’ respectively.

In the wake of increasing attention on dementia care, there is a growing awareness of these patients’ right to participate in decision-making processes concerning their own need for care and support. These are complex issues, especially if the wishes of the person with dementia may be potentially harmful or conflict with the beneficence and autonomy of informal caregivers [11]. Informal caregivers are crucial in the support of persons with dementia, also in decision-making processes and some may perceive it as a relief to leave decisions to others whom they trust [12, 13]. On the other hand, some may find it challenging, even offensive, that others make decisions on their behalf, even if it is close relatives [12–14]. Sustaining independence, agency and autonomy has been found to be important for persons with dementia in order to cope with a dementia condition and to experience dignity and quality of life [15–18]. Cahill [19] highlights that persons with dementia, too often are denied information and inclusion in decision-making processes considering their own care. It has also been documented how this often happens without properly assessing the person’s cognitive abilities [12]. The United Nations Convention on the Rights of Persons with Disabilities do however underline that the use of standardized tests to deny a person of legal capacity and human rights, based on mental capacity, is discriminatory and not permitted [20]. Rather, healthcare personnel are required to support the person to exercise legal capacity. Similarly, the WHO emphasize the need to empower persons with dementia to exercise legal capacity and to take part in the development of care structures [21]. Although the right to participate may be explicit parts of services’ ideology it may be challenging to pursue this ideal in practice [12]. However challenging it may be to pursue these ideals, we find it crucial to explore how to build structures within the health and care services that may increase patient empowerment and participation in decision-making processes.

The LIVE@Home.Path-trial (LIVE) (ClinicalTrials: NCT04043364, retrospectively registered) is a multicomponent, multi-disciplinary intervention aiming to support dyads, consisting of persons with dementia and their informal caregivers, to live safely and independently at home [22]. LIVE is an acronym for the main components in the intervention, that is: Learning; Innovation, Volunteers and Empowerment. A central part of the Empowerment-component is a dementia coordinator with the tasks of coordinating the LIVE-components and to ensure empowerment of the individual person with dementia throughout the LIVE-intervention process. This aims to ensure that the participants experience that the care and support they are provided are in line with their own perceived needs and wishes.

This present qualitative study is exploring the coordinator role before initiating the LIVE trial. Instead of making a strict, standardized description of the coordinators tasks, we wished to explore how the coordinators role and functions evolved in interaction with the participating dyads, consisting of persons with dementia and their informal caregivers. Further, we wished to explore issues concerning empowerment and patient participation throughout the intervention. The following research questions were addressed: 1) How do the participating dyads and the coordinators experience and describe the coordinator role and functions? 2) How can a coordinator contribute to the empowering of the person with dementia in decision-making processes – in everyday life and in planning present and future care and support?

## Methods

To answer the research questions, we used Patton’s [23] framework for qualitative program evaluation. This framework focuses on exploring variances in participants’ and program stakeholders’ experiences through dialog and attentive listening to their stories. A hermeneutic interpretive approach as described by Gadamer [24], was used to guide the qualitative interviews and the interpretive process. This approach focuses on how understanding comes forth through open and expecting dialogue. The principle of openness applies both to the interview situation between researcher and participant and to the interpretive dialogue between reader and text when analyzing interview transcripts [25]. User involvement in research increase the relevance and utility of scientific studies as it brings knowledge based on users unique experience into the research process [26]. A co-researcher with user experience as a long-time caregiver for a person with dementia were engaged in all parts of the study process: in designing the intervention and evaluation, developing interview guide, participating as a co-moderator in a focus group interview, in the analysis process, and throughout the writing process. These contributions proved useful in all phases of the study as he addressed critical remarks to our hypotheses; pointed at potential bottle-necks in the implementation concerning how participants organized their every-day life, how we as researcher best could avoid being an extra burden – while bringing his perspectives and horizon of understanding in the interpretive process.

### Study setting

Two specialist nurses, a woman and a man, were engaged as coordinators. Both were engaged at a municipal resource center for dementia and had long experience from nursing homes and home based care for persons with dementia. They also had thorough knowledge of available care and support. The resource center founded its work on the principles of person-centered dementia care as described by Kitwood [27]. The coordinators went on two initial home visits: the first to initiate contact and map out clinical data; the second, about a month after the first, to have an informal conversation with the dyads on “*What’s important to you?*” as well as discussing relevant support and aiding in application processes. Further, the coordinators contacted the dyads per telephone once per month to build a relation, evaluate the situation and discuss the way forward. The LIVE-components were discussed as possible solutions along with other existing support measures. After 6 months the dyads were offered a new home visit. In between contact points, the coordinators were available for contact if needed. Besides these established contact points, the coordinators were free to support the participating dyads as they deemed necessary. That meant they were free to judge to what degree they should support in application processes, increase telephone contact, provide extra home visits, arrange meetings with general practitioners and others involved and so on.

### Participants

We defined study participants as dyads consisting of the person with dementia and one informal caregiver. Inclusion criteria for the person with dementia were: having a dementia diagnosis; Mini-Mental-State-Examination score 15–24; living at home; ability to consent to participate in the study. The criteria for the caregiver participant was minimum one weekly face-to-face-contact with the person with dementia. Participants were recruited through the network of a municipal resource center for dementia and a local geriatric outpatient clinic. Eligible participants were informed of the study and included successively over a period of 2 months until a target of sixteen participating dyads were included. In a period of 6 to 9 months after the intervention started, six of the participating dyads of persons with dementia and their informal caregivers, plus three informal caregivers alone participated in qualitative interviews to evaluate the intervention. In addition, the two coordinators, along with their leader participated in a evaluative focus group interview [23]. In sum, we interviewed 18 persons to gather empirical data for this study.

See Table 1 for an account of the study participants.

**Table 1:** Study participants. (The coordinators and their leader’s age and gender are left out to safeguard anonymity)

ID	Role	Gender	Age	Interview type
A	Informal Caregiver	Female	68	Focus group
B	Person w/dementia	Male	67	Focus group
C	Informal Caregiver	Female	69	Focus group
D	Person w/dementia	Male	83	Focus group
E	Informal Caregiver	Female	65	Focus group
F	Person w/dementia	Male	65	Focus group
G	Informal Caregiver	Male	69	Dyad Interview
H	Person w/dementia	Female	75	Dyad Interview
I	Informal Caregiver	Female	75	Dyad Interview
J	Person w/dementia	Male	78	Dyad Interview
K	Informal Caregiver	Female	57	Dyad Interview
L	Person w/dementia	Male	69	Dyad Interview
M	Informal Caregiver	Female	71	Single Interview
N	Informal Caregiver	Female	59	Single Interview
O	Informal Caregiver	Female	57	Single Interview
P	Coordinator			Focus group
Q	Coordinator			Focus group
R	Coordinator leader			Focus group

### Data gathering

To answer the research questions, a hermeneutical approach, using qualitative interviews to explore the experiences of stakeholders in the project was chosen for gathering empirical data for this study. A basic principle within hermeneutical methodology is that our interpretation of the world around us will always depend on the interpreters’ background and pre-understanding [24]. The members of the research team had clinical experience as a medical doctor or registered nurse in care for persons with dementia in hospitals, nursing homes and home care. In addition, a co-researcher with user experience as an informal caregiver for a person with dementia was part of the research team. As research team, we had close dialogue with the coordinators throughout the intervention. Experiences made on these contact points and issues addressed were used to develop a semi-structured interview guide for the interviews. In addition to exploring experiences with the coordinator role, we also explored experiences with the LIVE-components. Results from this part of the interviews are not included in this study. Interviews were conducted in a period of 6 to 9 months after the study was initiated. Although we would have liked to recruit all 16 dyads, we realized throughout the intervention period that many of the informal caregivers had complex and demanding life situations. Some even had challenges in finding time for the coordinators’ home visits. Although these participants might have provided substantial insight we chose not to invite the most fragile of them – based on ethical considerations. Other dyads were out travelling throughout the period allotted for interviews.

Three persons with dementia participated in a patient education course for persons with dementia, in which their informal caregivers was welcome to join. The course was arranged by a local geriatric outpatient clinic and consisted of three two and a half hour sessions. Using opportunity sampling [23], these dyads participated in a focus group interview immediately after one of the course sessions. Thus we managed to arrange a group conversation between three dyads, exploring their experiences as participants in this study. Additionally, to explore various experiences, we used maximum variation sampling [23] to recruit three persons with dementia with their informal caregivers for single dyad interviews and three informal caregivers were interviewed alone in single interviews. These interview participants varied according to age; whether the dyads were living together or not; if and how much formal support they received; how long they had been living with dementia; and how much they had been in contact with the coordinators throughout the intervention period.

Finally, based upon the experiences of the project in total, along with initial analysis of the prior interviews, the gathering of empirical data were concluded with a focus group interview with the coordinators along with their leader. These were recruited as key informants [23], due to their central role in the study. In this interview, the co-researcher with user experience participated as a second co-moderator alongside an experienced co-moderator and the main interviewer. We are aware that our close collaboration and the growing relationship with the coordinators might have affected this focus group interview. Although we encouraged them to describe their personal view, we do not set aside the possibilities that this might have influenced their willingness to share critical remarks about the project. However, we experienced the interviews as open and honest and we believe that the relational interactions might have contributed to more openness around sensitive and emotional aspects.

### Ethical considerations

Informed consent is a basic principle when including human beings in research. When including persons with dementia in research, assessing the participants’ ability to understand information given might be a challenge. Often, this is solved by consent given by proxy from a family member or legal guardian. This, however, may exclude the person from having any say at all, concerning participation. Above, we have discussed the ethical and legal issues of depriving persons of their legal capacity based on mental or cognitive capacity [19, 20]. Hellström et al. [28] questions if provision of full information of a study’s content as a base for consent is actually possible for any person, regardless of cognitive functioning. In the case of persons with dementia, they describe ‘maximally informed consent’ as a useful concept and perspective. This entails adapting information to the individual person, repeating information, giving time to reflect and ask questions. Before inclusion to this present study, eligible dyads were informed about the main aspects of the intervention and asked to discuss participation with their family. During the first home visit, the coordinators, as specialist nurses, assessed the persons with dementia’s ability to consent, based on observations and clinical assessments. The participating dyads were thoroughly informed, together, about all aspects of the intervention and their right to withdraw at any time without further consequence. Information was carefully adapted, through dialogue, to ensure that the persons with dementia had understood the content. The persons with dementia and the informal caregivers both gave written consent to participate. On inclusion, the participating dyads were informed that they might be asked to participate in in-depth interviews as part of a six-month evaluation. They were informed that this was voluntary and that they had the right to decline this request without any consequences for their follow-up. Likewise, they were informed both when invited to participate in interviews and before the interviews started that this was still voluntary, that they at any time could ask to end the interview or that they afterwards had the right to ask for interview data to be deleted. They were also informed on procedures for data gathering and data treatment. As there is only one male and one female coordinator, they will consequently be referred to as ‘*she/her’* throughout the text, also in quotes from other participants, to protect their privacy. All interview participants consented to participate. The coordinators continued to follow-up all dyads after the study period was over. The study was approved by the Regional Committee for Medical and Health Research Ethics, Northern Norway (2017/1519 REK Nord).

### Data analysis

All interviews were audio recorded and transcribed, followed by initial analysis, successively. In this way, findings and horizons of understanding from prior interviews were assessed for potential further pursuit in the following interviews. For example if a participant emphasized new or unexpected themes, we would consider to address these themes in later interviews. Before the interview with the coordinators and their leader, initial analysis of the transcribed interviews were discussed by us as researchers – in search of themes to discuss in this final focus group interview. As a result, descriptions in the results section of general trends or variance among the participating dyads are based both on our interviews with the dyads and informal caregivers, and the coordinators descriptions of the intervention group. After all interviews were conducted and transcribed, they were analyzed separately by the members of the research team, as single texts, all interviews as a whole text and each interview as parts of the whole. Then, the research teams discussed their individual interpretations of the full body of texts in order to establish a shared understanding of the empirical data. This alternation between analyzing the interviews as single texts, as a whole and as parts of a whole characterized the analytical process, in line with hermeneutical methodology [24].

## Results

### The coordinator role and functions

The participating persons with dementia and their informal caregivers differed in age, symptoms, social network, as well as life situation in general. Accordingly, their need of support from their coordinator varied. The coordinators shared this view and described a high degree of variance in how much, and what kind of, support the dyads received throughout the intervention period. Some expressed being content with a brief telephone conversation once a month, while others used the monthly telephone calls for longer sessions of counselling, organizing various support measures or simply just to talk. It also varied how much the coordinators were contacted outside of the monthly telephone calls. This also had various reasons, ranging from how to prevent pills from falling on the floor while taking them out of the pill box, to arranging care solutions while the informal caregiver needed to travel, or the informal caregivers needing someone to talk to about minor or major concerns. Thus, the dyads’ perception of how their coordinator affected their everyday lives did also vary. Besides the home visits, it turned out to be only the informal caregiver who maintained contact with the coordinator, an aspect we will return to later in the results section. The first research question considered the coordinator role and functions. Although most of the dyads’ perspectives are described through the informal caregivers, the coordinator role and function affect the dyad as a whole and cannot be detached to only concern the informal caregiver. We found that the coordinators took three different functions in their relations with the dyads, to meet the variance in care needs. That is the function of *being a safety net*, *being a pathfinder*, and *being a source for emotional care and support*. In addition to the questions we had set out to answer, we found that good leadership and a trusting work environment was crucial for the coordinators to function in their roles.

#### The coordinator as a safety net

In general, all dyads expressed that they were satisfied with being part of the project. Some described how they experienced being at a point where the situation was stable, having the necessary support for the time being. One spouse described how she had struggled earlier, after her husband was diagnosed and they needed to find new solutions to cope with everyday challenges:

*I feel that perhaps it was too late to get help. The help we needed, we found out on our own and we had needed it when he got the diagnosis. (…) But if I’d had someone to play ball with from the start… (Participant O, informal caregiver)*

She emphasized how she had been in need of a coordinator at an earlier time, when her husband got the diagnosis. At the moment, she felt she had control and knew where to turn if she or her husband needed anything. However, she was aware that the situation might change and that it was good knowing the coordinator was there. Thus, the coordinator represented a safety net, that they could turn to when necessary, rather than an active contributor in their everyday lives. The spouse of a person who was recently diagnosed with dementia, and had not established any contacts within the system emphasized this dimension even stronger:

*As I say to (coordinator) when she calls: ‘at the moment, everything is fine, but thank you for being here and in the future when we perhaps are in need of help, we know someone will be there for us’ (Participant I, informal caregiver)*

Although some dyads perceived that there was little the coordinator could do for them at the moment, they emphasized the reassuring aspect of having a personal coordinator: *‘someone who knows us’(Participant I, informal caregiver),* ready to help if the situation should change. The coordinators confirmed this perception and emphasized how the regular contact helped them observe small changes in the dyads’ life situation as a whole. Further, the continuous documentation laid a foundation for future assessment of care provision by other instances, especially for the health administration, who are in charge of the formal assignment of public care and support. The coordinators’ leader underlined:

*Later, when they apply for something, nursing home or something, the documentation you do in the patient journal are good background information for the health administration.(Participant R, coordinators leader)*

The coordinators and their leader had a clear comprehension of the importance of observations, documentation and relational knowledge concerning these patients’ and informal caregivers’ current and future needs.

#### The coordinator as a pathfinder

One of the informal caregivers described how navigating the health administration and organizing the services for the persons with dementia were challenging and distressing: *I’ve felt that it’s incredibly difficult to find that thread, where do I start to wind? (M)* Several informal caregivers described this sense of not knowing where to start when seeking help. First, they found it difficult to identify and describe what the problem actually was. Second, due to limited knowledge about the municipal care and support services, they found it hard to identify and contact the right instances that might be able to help them. One spouse described how they experienced this in the time following her husband being diagnosed with dementia:

*It was a lot of phone calls, you know, I called the wrong persons. Then I got through to someone, I think it was at the social security or something, who gave me the number to (right person) and then things started to happen. (Participant C, informal caregiver)*

The dyads described how the coordinators in this situation played a more active part, helping them find and acquire good solutions. Based on the dyads’ needs and wishes, this help ranged from giving advice on how to solve everyday challenges, providing simple information on where to find application forms and, in some cases, to coordinate collaboration between services. As concrete examples, the coordinators were counselling representatives of the home care service on how to interact with the person with dementia when preparing his or her breakfast. The coordinators also arranged a meeting place for representatives of the daycare center and the short-term nursing home ward for developing a cooperation ensuring continuity of collaborative routines when the person with dementia had a respite stay at the nursing home. Thus, their support focused on the needs of the persons and were not confined to the LIVE-components. One informal caregiver, who had a full time job and did not live with the person with dementia, described how the coordinator in this way helped them through a challenging life situation:

*To me, it’s been very important (the coordinator’s help), because it’s a lot of work and many things to follow up, you know. It’s appointments with the general practitioner, the ophthalmologist, then he has some skin problems, so it’s the dermatologist... Then you need to stay in touch with the homecare services, and remember to buy medicines at the pharmacy and remember this and that and then less and less is working at home. So I felt that having one person to relate to has been very good for me. To kind of see some steps ahead, you know, she can say ‘I think you might need… and we might as well apply right away’ (…) If I feel that we need some more help, I talk to (coordinator), I don’t talk to anybody else. (Participant K, informal caregiver)*

The description illustrates some of the vast complexity in caring for a person with dementia. For this participant, the coordinator had, in many ways, taken over coordination of the care and support as a whole. Although the informal caregiver experienced a heavy burden, the coordinator had an overview over the total situation and did most of the communication with all the different healthcare providers involved. Thus, we have found the metaphor ‘pathfinder’ as an appropriate concept describing this crucial coordinator function of keeping a survey of the dyads’ situation as a whole and leading them on the way to possible means of support. In conversation with the coordinators concerning the time and resources they used on this kind of work, they claimed that in total it did not consume much time. They also experienced that it helped provide purposeful support, thus reducing waste of resources within the health and care services. However, they emphasized how they primarily perceived this consideration as a means to safeguard the persons with dementia:

*No person with dementia wants to get a lot of people into their homes, nor a lot of services. They want exactly what they need and nothing more. (Participant P, coordinator)*

The coordinators perceived that most participating persons with dementia were reluctant to receive support, especially within their home, and that they wanted to keep support at a minimum. The coordinators’ knowledge of the available support and how to attain it, and their thorough knowledge on the dyads’ life situation as a whole, enabled them to recommend care and support that was individually adapted. Throughout their follow-up process, the coordinators had slightly differing support approaches when applying for services, − a process that at times might be quite strenuous due to a complex administrative system. While one of the coordinators aimed at informing and guiding on available, adequate services and how to apply, the other coordinator also helped writing application forms as well. Although the practical work did not differ much, the perceptions of how this worked differed, as illustrated by this informal caregiver:

*(The coordinator) can say “application forms for this and that is on the internet or I can send it in a mail if you have difficulties with the net” or something like that. But it’s not like I can say “we need this and that…” you know, to simplify the application process. If you’re used to orient on the internet, it’s actually nothing she’s said that I hadn’t found out on my own. But still, it’s good to have her, because you can kind of check with her “can I do this or that, does it work like this?” but still, you have to move on, on your own. (Participant G, informal caregiver)*

Although this informal caregiver found it hard to handle this challenging procedure himself, he still found support in being able to seek advice from the coordinator. Overall, despite differences in perceptions, most dyads experienced the coordinators as someone who helped them find and consider different possibilities, helping them cope in their everyday lives.

#### The coordinator as a source for emotional care and support

Some informal caregivers also found the coordinators contributions in terms of emotional care and support vital. The need for this kind of care might be due to a general high level of distress and vulnerability, experiencing a crisis during the project period or simply dealing with the multiplicity of small everyday challenges. One spouse described how she almost despaired when they had come home from a travel and she couldn’t reach the coordinator.

*I couldn’t reach her that day, I felt that I almost (grips around her throat and chokes)… but then I got through and then it’s half an hour and then all problems are solved… plus some I didn’t knew I had*. *(Participant A, informal caregiver)*

The remark illustrates how the coordinator may be seen as someone who help making everyday challenges and suffering endurable. Further, it indicates how the coordinator might serve as a problem-solver over the telephone. The coordinator did not only answer to the dyads’ everyday challenges, she also brought to light related issues that the dyads did not think about themselves, as well as presenting possible solutions to them. Other informal caregivers emphasized the benefit of having the coordinator to contact, also for emotional support:

*If I’ve been desperate, you know, this and this has happened. Then I call and talk to her and usually, she has a good idea, so it’s very good talking to her. (Participant M, informal caregiver)*

The importance of having someone to talk to one-to-one, who knew them and their current life situation were addressed by several informal caregivers. Combined with input on how to cope with their present challenges, these opportunities to talk to a coordinator that listened and supported them was crucial for them. One of the coordinators described how they experienced this vital aspect as well: *“I feel that I often am that ‘container’ that listens and confirms’ (Participant P, coordinator).* A daughter, caring for her father with dementia described how essential it was for her to be seen and taken care of herself, by the coordinator:

*I get to talk to someone who perhaps has another view and some knowledge, because I know very little about this myself. (…) And someone who tells me: ‘you know, you need to take a time-out!’ I need that someone tells me (…)I feel that it’s not only one-way communication, that it’s only me who calls and ask if anything comes up, but that they actually make contact… (Participant N, informal caregiver)*

Consequently, some informal caregivers experienced the benefit of getting support, but also a little push, to help them make difficult decisions and taking care of themselves in times when caring for the family member with dementia became all-consuming. Some of the dyads also found themselves in the midst of challenging life situations not related to the dementia condition itself. One coordinator experienced how a complex family conflict created an extra burden for the dyad. Although there was little the coordinator could do about the issue at hand, she still found it crucial to listen, support and acknowledge the dyads’ attempt to cope with the situation:

*At least it has become (natural), because it’s what she needs. (…) And she does a lot of good things (…) so she kind of needs someone who listens and supports and say ‘this was good’(Participant P, coordinator)*

Although peripheral to the direct support of the person with dementia, the coordinator recognized how the surroundings affected the everyday life of the person with dementia. Thus, aiding to relieve the tense situation within the family would be beneficial for the well-being of the person with dementia.

#### Emphasis on trust based working conditions for the coordinators

Although not an initial focus of inquiry, the coordinators underlined the importance of having a trust based relationship to their supervisors to be able to fulfill their tasks as coordinators:

*From the first moment the project was presented, we’ve had full support and recognition on this as something to prioritize and spend time on. And that has made it easy to get going and I think that is important. About having a leader and get time to do the tasks. We haven’t spent time on defending why we have to take this and that home visit, you know. And that’s not to be taken for granted. (Participant Q, coordinator)*

An important aspect for the coordinators in order to be able do their tasks was to get recognition and trust from their superiors on their priorities and choices of action. Likewise to get support and supervision in challenging situations:

*I sometimes get a bad conscience and a little stomach ache… but then, I am quite good at talking about all my feelings (laughs). But to me it’s important, in this job, to have good colleagues that I can talk to, because I don’t talk about it at home. And I think that it’s quite natural to have these feelings, so I don’t really think it’s a problem. Perhaps it’s a little barometer, I don’t know. I use (other coordinator) a lot, and (leader). Good supporters. (Participant P, coordinator)*

The coordinators described how they experienced feelings of unease and emotional distress as a part of their work as a coordinator. Although straining, the coordinators did not describe this as a negative thing: rather the negative emotions were perceived as an indicator of the ability to build empathy and get involved in a relationship with the participant. Equally important though, was the emotional and professional support from co-workers and leaders. The leader agreed and confirmed how this kind of support should be prioritized:

*Let them get the chance to reflect and mature as coordinators, I think a lot about that. I want to handpick personnel, you know. It is something very serious about being a coordinator, because you have such an impact on peoples’ lives and you’re not to be too easy on it either.(Participant R, coordinators leader)*

The mutual trust and recognition between the coordinators and their leader seemed to be crucial for the feasibility of the trial. This statement further reveals a serious awareness of the gravity of the coordinators work. Both coordinators and their leader described a determinate focus on how to support the individual, regardless of if and how this support was measureable or might fit into goal attainment scales.

### Empowering the person with dementia in decision-making processes

Considering the second research question, we found that meeting the person with dementia alone during the first home visit was perceived crucial for the coordinators to get an impression of the persons’ values and preferences. However, the coordinators found it challenging to maintain an empowering relation with the person with dementia throughout the study period.

#### Challenges in establishing relationship with the person with dementia

During the first home visits, the coordinators had one-to-one conversations with the participants living with dementia, based on the question ‘*What’s important to you?’* in addition to mapping out clinical data. The second home visit solely focused on building a relation with the dyads. Here, they emphasized involving the persons with dementia in the dialogue and getting their views on the themes of discussion. This was deemed valuable to be able to get an impression of the person and the persons’ values and preferences.

“*You get to hear a lot about their lives and experiences and you get to know what’s important to them, and what has been important to them. … You sort of get to know their pulse a little… You get an image of who this is. If I ask ‘what’s important to you, now?’ it’s not always that easy to get an answer.” (Participant P, coordinator)*

In this statement we also see a recognition of how the answer to the question of ‘*What’s important to you’* not necessarily is found by asking directly, but rather is hidden somewhere within the participants’ narratives. However, during the continuous follow-up by telephone, which was intended to sustain the contact with both members of the dyads, the coordinators found it challenging to uphold direct contact with the persons living with dementia. The frequency of once a month and contact by telephone made this challenging, especially because of the person’s illness-related memory problems:

*Calling to someone who has dementia… It’s not a good way to communicate, you know. And building a relation to someone with dementia and meeting once every third month or half-a-year is not good either. Because the person with dementia won’t remember you and will get anxious every time. … It might have worked in some cases, but it’s not right towards the person with dementia. (Participant P, coordinator)*

Thus, the monthly follow-up by telephone turned out to be only between the coordinators and informal caregivers and none of the persons with dementia initiated contact with the coordinators themselves. Most of the persons with dementia described experiences with support that were facilitated as part of the follow-up and one of the persons with dementia described it like this:

*We’ve got this and that, so it’s been great… comes home and we sit and talk, so it’s been good. She’s coming next Friday. (Participant F, person with dementia)*

This person had a clear view of who the coordinator was and valued the home visits and the support they had received through their coordinator. The other participating persons with dementia, did however describe little conception of who their coordinator was and what their exact role was. The following conversation between interviewer (I), person with dementia (P) and informal caregiver (C) illustrates this issue.

I*: Do you feel that you have a relation to (coordinator)?*

*P: I really can’t say that.*

*C: Do you know who we are talking about?*

*P: No… I know I have met her…*

*C: Many times, she’s been visiting us several times.*

*P: You know, I don’t recognize all those actors out there.*

*…*

*C: I think that if you’d seen her, you would have recognized her.*

*P: I guess*

*(Participants K/L, informal caregiver /person with dementia)*

It must be underlined that the person in question throughout the interview had little difficulties in recollecting details and aspects from his everyday life. Some persons with dementia expressed a remembrance of the meetings and the coordinators as nice persons, but they expressed little perception of the coordinators as someone who helped and supported them in any way. Thus, although the persons with dementia took active part in the interview conversations and shared their experiences of the support they received in general, they had little substantial response regarding their perception of the coordinator role.

## Discussion

Concerning the first research question, about the coordinators’ role and functions, the participating informal caregivers described how they experienced three different coordinator functions. First, they described how the coordinators served as a means of safety and comfort. Although some perceived that there was not much the coordinator could do at the time, they appreciated that there was a person who knew them and their situation, who was ready to help them if needed. Second, the coordinator served as a co-planner and organizer of care and support. This function was flexible and ranged from giving basic information on available care and support to more or less taking over coordination of the many health and care services involved. Third, the coordinator served as valuable emotional support and care for the informal caregivers in times of distress. These three functions built upon each other. Based upon the relation and knowledge of the total situation gained through the basic phone calls, support could be more individually adapted, when needed. Further, the building of a strong relation made a foundation for providing adequate emotional support. In total, getting to follow up the persons with dementia and their caregivers over time, with a view on the whole situation, laid the ground for a wide perspective making holistic care and support possible.

In line with the encouragement from Iliffe et al. [10] we will establish these three functions as crucial components for care and support coordination for this group to function. Further, we find the concept of ‘fluidity’ to be adequate also to describe the internal relation between the components described. They overlapped and the borders between these functions were at times fluctuating. The coordinator functions as described should therefore not be seen as distinct tasks to be pursued as such, but as functions that should get room to arise in the interaction between the coordinator and the dyad. Moving away from task-oriented standardization is supported by care philosopher Kari Martinsen [29]. She asserts the importance of meeting the other, in this case the dyad, with openness and wondering. This requires a fine balance between managing to see past the immediate without invading the others’ private sphere. The coordinators’ different approaches to support in application processes exemplifies how complex it may be to find this balance in practical approaches. It also illustrates how relatively small differences in approaches may affect participants’ assessments of the support they are receiving.

The coordinators and their leader underlined the importance of mutual trust and good relations to ensure the necessary flexibility to provide adequate follow up of the participants. Equally important, they described a need to ensure professional and emotional support for the coordinators. These descriptions also involved the coordinators’ recognition and acknowledgement of their own vulnerability in meeting the dyads. Martinsen [29] emphasize how emotional involvement calls for increased attention, making room for good professional judgments of what is at stake in the situation. She makes a clear distinction of this kind of involvement as opposed to sentimentalism. Sentimentalism, she claims, might emerge as a result of suppressing emotions for the sake of professionalism. Within leadership theory, the importance of good leader-employee-relationships and mutual trust has shown to have high effect on productivity as well as the employees’ mental health [30]. Kitwood [27] emphasize the crucial role of leadership and work environment within person-centered dementia care. Likewise, as the concept of person-centered care has been further developed into a theoretical nursing practice framework, the aspect of work environment and leadership has been established as integral parts. McCance and McCormack [31] describes how personal prerequisites in the healthcare personnel, such as competency, skills, values and commitment as well as structural aspects in the care environment, including supportive systems, staff relationships and power sharing are crucial to succeed with this kind of work. Their framework also includes ‘sympathetic presence’ as a prerequisite, again underlining the need for emotional involvement in order to do professional judgments. Although these aspects were not initially addressed in this present study, we found clear similarities between coordinators’ descriptions of the prerequisites to be able to perform their tasks and the prerequisites described within the person-centered framework. Similarly, a qualitative review of stakeholders’ perspectives on coordinating care in dementia emphasize the importance of support for the coordinators [32].

Considering the second research question, on empowerment of the persons with dementia, we found that this was a challenging aim to pursue directly within the structures of the intervention. The initial conversations between coordinator and person with dementia alone, in a trusting environment, were crucial for the coordinators to get to know the person values and preferences. The remark about finding an answer to the question *‘What’s important to you’* in the persons’ narratives illustrate the importance of finding room for such conversations between coordinator and person with dementia. An open approach in these conversations may further help the coordinator gain insight in the persons’ rhythms of daily life at home [33]. However, we found that it proved challenging for the coordinators to pursue this objective throughout the intervention period. Both the frequency and means of contact was perceived as suboptimal. Due to these challenges, we have increased the contact points and included a process of Advance Care Planning (ACP) in collaboration with the persons’ general practitioners in the main LIVE study [22]. Further, we believe that the persons with dementia might benefit indirectly through the empowering support of the informal caregivers and the support measures instigated by the coordinators. We acknowledge that we should have investigated more thoroughly whether phone calls could have been feasible to maintain contact, at least with some of the persons with dementia.

The issue of ensuring genuine patient participation for home dwelling persons with dementia is complicated. In a multi-case study of ten cases, Smebye et al. [12] describes how healthcare personnel and informal caregivers may engage persons with dementia in shared everyday decision-making, such as choosing among activities, what to eat or when to shower. As such, the informal caregivers play a crucial role in empowering the persons with dementia and are central collaboration partners for healthcare personnel in general when decisions on care and support are made. On the other hand, Smebye et al. [12] also describe instances of not involving the person at all or pseudo-autonomous decision-making. The latter defined as cases where the person with dementia was not adequately informed and decisions were based on mere assumptions about the persons’ values and preferences. Similarly, Taghizadeh Larsson and Österholm [34] investigated 24 qualitative articles on decision-making for persons with dementia. Although they found examples where the wish of persons with dementia were respected, exclusion of the person with dementia in decision-making processes was reported as the most frequent finding. Advance Care Planning (ACP) as a repeated process to plan for future care and treatment in line with the patients’ values and preferences has been increasingly common within dementia care [35]. Also within this concept, exclusion of the person with dementia, often without giving account for why, was the most frequent finding in a systematic review, including 30 articles [36]. As the available support for persons with dementia is getting more specialized and individually adaptable, the amount of decisions being made concerning care and support is increasing accordingly. The informal caregivers’ role in supporting the persons with dementia in decision-making processes should not be underestimated.

Still, to safeguard the human rights [20] of persons with dementia to take active part in decision-making processes, these processes needs to be adapted for this to take place. In a meta-ethnography on agency in dementia, Bosco et al. [37] reveals how acknowledging the persons as active agents and helping them maintain positive views of their abilities are important first steps to help them maintain autonomy. Further, they describe how persons with dementia can be supported in building strategies for making decisions on their own, such as by breaking decisions down to smaller units or using simple aids, such as a diary. In a recent study, we explored home-dwelling persons with dementia’s perception on different support measures. We found that, when given time, space and adapted explanations, all participants reflected on hypothetical future scenarios and how support measures could be adapted to suit their needs, even when they had no prior knowledge of the measure [14]. Similarly, Smebye et al. [12] claims that the question of patient participation should not be *if,* but *how,* in line with the demand from the United Nations to support the patient to make decisions regarding their own care [20]. We recommend an increased emphasis on these aspects, both in the training of health care personnel and in the counselling of informal caregivers for persons with dementia, in line with the recommendations of the WHO [21]. In this way, we hypothesize that the coordinators through their counselling and support of the informal caregivers may obtain increased empowerment for the persons with dementia.

### Implications for practice and further research

Based on the experiences in this study, we believe the framework for follow-up as presented is a feasible starting point for supporting informal caregivers for persons with dementia living at home. We also recommend that this follow-up start at an early time after a dementia diagnose has been set, both in order to build a strong relation to the dyads, but also to get necessary support in order at an early time. In a systematic review, Backhouse et al. [32] points to a general consensus of the importance of offering care coordination at the point of diagnosis. Based on the three functions we identified and the fluid borders between them, we recommend a flexible approach, with a trusting work environment where the coordinators are given space to distribute their time according to the shifting needs of the participating dyads. This also entails a supportive work environment where the coordinators are given room to reflect, share difficult emotions and mature in their roles.

Informal caregivers are crucial in the daily care for persons with dementia and they are deeply affected by the dementia condition [7, 38]. The concept of ‘relational autonomy’ [39, 40] highlights this role and emphasize the inclusion of informal caregivers and the persons’ wider social context in decision-making processes. This should however not exclude the persons with dementia from participation where this is possible. Non-involvement or pseudo-autonomous decision-making by informal caregivers or healthcare personnel are serious moral and legal issues, denying the patient of basic human rights and depriving them of their status as a person [19]. Conversely, retaining autonomy is crucial for persons with dementia to sustain quality of life and dignity [15, 16, 18]. Additionally, although sometimes difficult to measure, we argue that focused attention on patient empowerment – anchored in an ontological perspective of caring [29] as well as in human rights [20, 21], naturally should include, and not exclude, persons with dementia. In the further development of the LIVE-trial we attempt to strengthen this perspective with more home visits and initiating a process of ACP involving the persons’ general practitioners. This entails involving family members, general practitioners and other health care personnel in repeated, structured conversations, started at an early point in the dementia progression, where the person with dementia has the opportunity to share their wishes and values [22]. In this way, we aim to strengthen the inclusion of persons with dementia in decision-making processes, also on medical questions. This will also comply with the suggestions of starting ACP-process early and move the concept beyond questions of end-of-life care [35, 36, 41]. Informal caregivers of persons with dementia are subject to a high burden [7] and making difficult decisions on behalf of the person may increase emotional distress [42]. Healthcare personnel are requested to support persons with dementia to participate in decision-making processes [14, 20]. We recommend them to share this perspective with informal caregivers to increase patient participation in everyday decision-making. However, these are complex issues and the knowledge of how to enhance patient participation for this group is limited. We suggest an increased focus on this issue in future research. In line with recommendations from the WHO, we suggest that research upon these matters include the involvement of persons with dementia and their informal caregivers [2, 43].

### Strengths and limitations

This study is based on interviews with eighteen stakeholders in an intervention with a relatively small number of participants. One of the reasons for not recruiting more of the persons with dementia who were part of the intervention in the interviews, was that we early got a clear apprehension that they were little involved in the interaction with the coordinators. Thus, we assumed that they might give limited contribution to answering the research questions. When asking about the coordinator follow-up in the interviews, the informal caregivers often took the word while the person with dementia showed signs of uncertainty. In order to safeguard the persons’ integrity, in situ, the persons with dementia’s views on this theme were not further pursued, except in the situations referred in the results part of the study. In retrospect, we realize that the issue of limited patient participation should have been more closely explored in the interviews and that we should have put more effort in exploring the experiences of the persons with dementia. A co-researcher with user experience was part of the research team throughout all faces of the study.

## Conclusion

This study aimed at exploring the role and function of a coordinator for persons with dementia and their informal caregivers. Within systematic frames for a minimum frequency of contacts and overarching tasks, the coordinators were given quite free reins on how to adapt the follow up for the individual. We found that the coordinators fulfilled three functions in relation to the caregivers. That is, the function of representing a safety net; of being a pathfinder; and as a source for emotional care and support. To be able to fulfill their role as coordinator, a trusting working environment was emphasized. Further, we found that it was challenging for the coordinators to establish genuine relations in order to empower the persons with dementia in decision-making processes. This indicates a need for frequent meetings between coordinator and person with dementia and will be pursued in the main study of the LIVE-trial. Further research on how to establish sustainable, genuine patient participation on a broad scale for this group is required.

## Supplementary Information

**Additional file 1.**

**Additional file 2.**

## Data Availability

All empirical data (sound recordings and transcriptions) are stored on a secure server at the University of Bergen. Due to ethical concerns, the material is not publicly available. Data will be deleted at the end of the project.

## Abbreviations

ACP: Advance Care Planning
LIVE: LIVE@Home.Path-trial
WHO: World Health Organization
