# Supplementary material for: The compound role of a coordinator for home-dwelling persons with dementia and their informal caregivers: qualitative study
Source: BMC Health Serv Res. 2020 Nov 16;20:1045. doi: 10.1186/s12913-020-05913-z (PMC7670600; doi:10.1186/s12913-020-05913-z)
Supplement: Supplementary file 1 — Additional file 1. [file 12913_2020_5913_MOESM1_ESM.docx]

**Interviewguide – Coordinator/leader**

**Can you describe the project you have taken part in?**

What is it about for you? Why did you accept to join? Expectations? Doubts?

**How do you experience the relation to the participants?**

The persons with dementia? The informal caregivers? How do the relations appear? What characterize a good relation? What characterize less good relations?

**Can you say something about how you have approached the variety of needs and resources in the participants?**

‘What’s important to you?’-focus? Variance in needs – support, counsel, services, coordination?

**What do you think about the structure of the project?**

The coordinator-role? LIVE-components? Follow-up – home visits, phone calls? Available resources? Frames and possibilities related to project objectives?

**What experiences have you done in cooperating with other instances as part of follow-up?**

Who/what/how/why? How has this been? What has your role been? How to balance being a shortcut and a detour to the services? Adminitration vs services?

**How do you experience the participants’ expectations?**

How do you experience meeting expectations? What characterize challenging expectations? How can this be solved?

**What experiences do you have on the LIVE-components?**

What attitudes do the dyads have? What impact has follow-up over time – giving time to think about solutions? How do the participants describe their experiences?

**What do you think about the information you got ahead of the project?**

What was good? What was missing? Did you have unanswered questions? Did you feel ready to go ahead with the tasks? Follow-up underway? Structures?

**How did you experience using questionnaires?**

Questions? Answering options? Setting? Utility value for follow-up? Uncomfortability/challenging questions? Positive? Questions opening for conversations you would have got otherwise? Did you miss any questions?

**What is the most important experience to share with future coordinators?**
