# Supplementary material for: The compound role of a coordinator for home-dwelling persons with dementia and their informal caregivers: qualitative study
Source: BMC Health Serv Res. 2020 Nov 16;20:1045. doi: 10.1186/s12913-020-05913-z (PMC7670600; doi:10.1186/s12913-020-05913-z)
Supplement: Supplementary file 2 — Additional file 2. [file 12913_2020_5913_MOESM2_ESM.docx]

**Interview-guide – Dyads/Informal caregivers**

**Can you tell me about the project you have participated in?**

What is it about for you? Why did you want to take part? Expectations? What information did you get before participating? Doubts about participating?

**What has been important for you during participation?**

What has participating meant for you? In everyday life? In general? Has something changed? What? How?

**How do you experience the relation to the coordinator?**

Limitations/possibilities? Safety/insecurity? Availability? Degree of follow-up?

**Do you have any experiences with… (one and one of the LIVE-components)**

Why/why not? How? How did it work? Did you have any of these beforehand? Had you been thinking about any of these things beforehand? What made you consider this? Experiences? What can be relevant in the future?

**How do you feel participation has influenced you as persons?**

As couple/family/dyad? Has the relationship to others (family/friends) changed?

**Is there anything you have missed in the project?**

What could have been done differently? Has anything been unclear?

**How did you experience filling out questionnaires?**

Questions, answering options, setting? Questions/themes you are missing?

**What do you think should be the next step in developing care and support for you and/or others in the same situation?**

Can you describe 1-3 measures that could have made everyday life easier?
